# CUZD1 is a critical mediator of the JAK/STAT5 signaling pathway that controls mammary gland development during pregnancy

**DOI:** 10.1371/journal.pgen.1006654

**Published:** 2017-03-09

**Authors:** Janelle Mapes, Quanxi Li, Athilakshmi Kannan, Lavanya Anandan, Mary Laws, John P. Lydon, Indrani C. Bagchi, Milan K. Bagchi

**Affiliations:** 1 Department of Molecular and Integrative Physiology, University of Illinois Urbana/Champaign, Urbana, IL, United States of America; 2 Department of Comparative Biosciences, University of Illinois Urbana/Champaign, Urbana, IL, United States of America; 3 Department of Molecular and Cellular Biology, Baylor College of Medicine, Houston, TX, United States of America; National Cancer Institute, UNITED STATES

## Abstract

In the mammary gland, genetic circuits controlled by estrogen, progesterone, and prolactin, act in concert with pathways regulated by members of the epidermal growth factor family to orchestrate growth and morphogenesis during puberty, pregnancy and lactation. However, the precise mechanisms underlying the crosstalk between the hormonal and growth factor pathways remain poorly understood. We have identified the CUB and zona pellucida-like domain-containing protein 1 (CUZD1), expressed in mammary ductal and alveolar epithelium, as a novel mediator of mammary gland proliferation and differentiation during pregnancy and lactation. *Cuzd1*-null mice exhibited a striking impairment in mammary ductal branching and alveolar development during pregnancy, resulting in a subsequent defect in lactation. Gene expression profiling of mammary epithelium revealed that CUZD1 regulates the expression of a subset of the EGF family growth factors, epiregulin, neuregulin-1, and epigen, which act in an autocrine fashion to activate ErbB1 and ErbB4 receptors. Proteomic studies further revealed that CUZD1 interacts with a complex containing JAK1/JAK2 and STAT5, downstream transducers of prolactin signaling in the mammary gland. In the absence of CUZD1, STAT5 phosphorylation in the mammary epithelium during alveologenesis was abolished. Conversely, elevated expression of *Cuzd1* in mammary epithelial cells stimulated prolactin-induced phosphorylation and nuclear translocation of STAT5. Chromatin immunoprecipitation confirmed co-occupancy of phosphorylated STAT5 and CUZD1 in the regulatory regions of epiregulin, a potential regulator of epithelial proliferation, and whey acidic protein, a marker of epithelial differentiation. Collectively, these findings suggest that CUZD1 plays a critical role in prolactin-induced JAK/STAT5 signaling that controls the expression of key STAT5 target genes involved in mammary epithelial proliferation and differentiation during alveolar development.

## Author summary

In the mammary gland, genetic circuits controlled by the hormones, estrogen, progesterone and prolactin, act in concert with pathways regulated by members of the epidermal growth factor family to orchestrate growth and morphogenesis during puberty, pregnancy and lactation. We have identified CUZD1 as a novel mediator of prolactin signaling in the steroid hormone-primed mouse mammary gland during pregnancy and lactation. *Cuzd1*-null mice exhibited a striking impairment in ductal branching and alveolar development during pregnancy, resulting in a subsequent defect in lactation. Administration of prolactin failed to induce proliferation of the mammary epithelium in *Cuzd1*-null mice. Protein binding studies revealed that CUZD1 interacts with downstream transducers of prolactin signaling, JAK1/JAK2 and STAT5. Additionally, elevated expression of *Cuzd1* in mammary epithelial cells stimulated phosphorylation and nuclear translocation of STAT5. CUZD1, therefore, is a critical mediator of prolactin that controls mammary alveolar development.

## Introduction

In the mammary gland, development of an extensive ductal network during puberty and formation of lobuloalveolar units during pregnancy are critical events required for lactation. These complex developmental processes are regulated by a variety of signaling cues, including the steroid hormones 17β-estradiol (E) and progesterone (P), the peptide hormone prolactin (PRL), and the epidermal growth factor (EGF) family of growth factors [1]. During pregnancy and lactation, E, P, and EGF family members act in concert with PRL to induce alveologenesis, a process in which ductal epithelial cells undergo extensive proliferation and secretory differentiation [2,3].

Circulating levels of PRL rise during pregnancy and promote proliferation and differentiation of the mammary epithelium in preparation for lactation [4–7]. The prolactin receptor (PRLR) is a trans-membrane protein belonging to the cytokine receptor superfamily [8]. Binding of PRL to PRLR triggers signaling events through the JAK/STAT5 pathway [9,10]. Janus tyrosine kinase 1 (JAK1) and janus tyrosine kinase 2 (JAK2), associated with PRLR, are rapidly phosphorylated upon PRL binding. Signal transducer and activator of transcription 5 (STAT5), which is phosphorylated following JAK activation, undergoes dimerization and localizes to the nucleus [9,11–13]. The tyrosine phosphorylation of STAT5 is essential for DNA binding and transcriptional regulation [10]. Activated STAT5 binds directly to the GAS motif (TTCnnnGAA) at target genes to regulate their transcription and promote proliferation and/or differentiation of the mammary epithelium during distinct phases of mammary gland development [11]. It was reported that PRL signaling through JAK2/STAT5 activates cyclin D1 transcription and nuclear accumulation in proliferating mammary epithelial cells [14]. Furthermore, STAT5a has been shown to regulate transcription of other mitogenic factors, such as the EGF family member epiregulin [15,16]. Terminal differentiation of the mammary gland is defined by the expression of milk protein genes in preparation for lactation. STAT5 controls the expression of several of these genes, including whey acidic protein (*Wap*) and β-casein (*Csn2*), to induce functional differentiation of the alveolar epithelial cells [11,12,17–20]. These observations established that STAT5 signaling is essential for proliferation and differentiation of the mammary gland.

Ample evidence exists to suggest integrated effects of PRL and EGF receptor (ErbB)-mediated signaling pathways during mammary gland development. Binding of specific EGF ligands induces differential heterodimerization of ErbB family receptors to stimulate specific intracellular signaling pathways, thereby accounting for the varied effects of an activated receptor. Upon EGF administration, STAT5 is activated to a similar degree as seen with PRL treatment [3,20]. Furthermore, active ErbB4 was shown to induce phosphorylation of STAT5 in the mammary epithelium [21]. *ErbB4*^(-/-)^ mice exhibit disrupted alveologenesis and a dramatic reduction in the expression of *Wap* and further investigation revealed that STAT5 phosphorylation is lost [21]. These findings pointed to a possible link between signaling via EGF family receptors and STAT5 activation to control alveolar proliferation and differentiation, although the precise molecular basis of this crosstalk remains unclear.

This study reports that CUZD1 is a novel mediator of PRL and EGF signaling in mammary epithelial proliferation and differentiation during pregnancy. This protein, also known as ERG1, Itmap1, or UO-44, was originally identified in our laboratory as an E-regulated gene in the rodent uterine epithelium and later reported in other tissues [22–25]. CUZD1 contains a zona-pellucida (ZP)-like domain and two tandem CUB (Complement subcomponent /C1s, Uegf, Bmp1) motifs (S1A Fig). There is presently little information concerning the functional significance of these motifs, although their presence is often noted in molecules involved in developmental processes [26,27]. The mouse *Cuzd1* gene shares strong sequence identity with its human ortholog, indicating functional conservation across species (25). Using a *Cuzd1*^(-/-)^ mouse model and a combination of *in vivo* and *in vitro* approaches, we investigated the molecular pathways that are controlled by *Cuzd1* in the mammary gland and uncovered a novel mechanism linking CUZD1 to the PRL and EGF family growth factor signaling pathways that guide epithelial proliferation and differentiation in the mammary gland during pregnancy.

## Results

### CUZD1 controls alveolar morphogenesis during pregnancy and lactation

**Fig 1:**
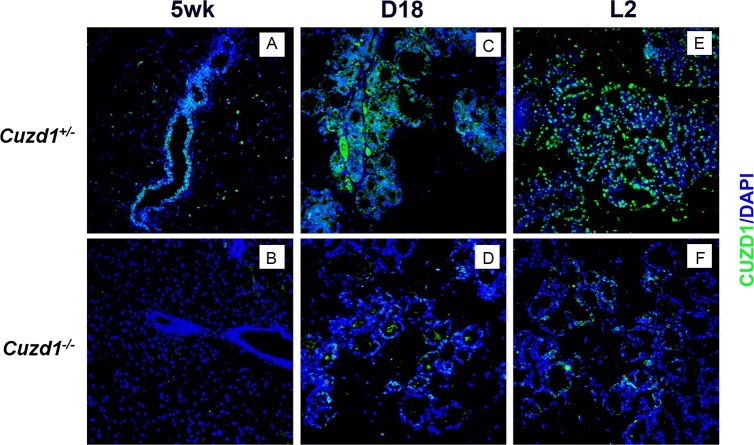
Analysis of the spatio-temporal expression of CUZD1 in the mammary glands during development. Mammary sections were obtained from *Cuzd1*^(+/-)^ and *Cuzd1*^(-/-)^ mice at puberty (5 weeks, a and b), late pregnancy (Day 18, c and d) and lactation day 2 (L2, e and f) and subjected to immunofluorescence, using rabbit polyclonal antibodies against mouse CUZD1. Magnification 20x.

We examined the expression of CUZD1 in the mammary glands of *Cuzd1*^(+/-)^ and *Cuzd1*^(-/-)^ mice at different stages of development: pubertal (5 weeks), late pregnancy (D18) and early lactation (L2). Immunofluorescence (IF) analysis of CUZD1 revealed no detectable expression in mammary tissue of *Cuzd1*^(-/-)^ mice during development (Fig 1B, 1D and 1F). CUZD1 was detected in the developing ductal epithelium of *Cuzd1*^(+/-)^ mice at puberty (Fig 1A). CUZD1 immunostaining was also observed in both cytoplasmic and nuclear compartments of the ductal and alveolar epithelial cells of *Cuzd1*^(+/-)^ mammary glands during alveologenesis at late pregnancy (Fig 1C). Prominent nuclear staining was seen during lactation (Fig 1E), indicating that CUZD1 may play a critical role during mammary gland development, particularly during pregnancy and lactation.

To investigate the functional role of CUZD1 in mammary gland development, we created *Cuzd1*^(-/-)^ mice in which this gene is deleted from the mouse germ line by homologous recombination using mouse embryonic stem cells (S1B Fig). The efficiency of gene deletion was confirmed by PCR analysis of genomic DNA (S1C Fig) and northern blot analysis of *Cuzd1* mRNA expression (S1D Fig). The *Cuzd1*^(-/-)^ females were fertile and delivered normal size litters. However, the majority of pups from *Cuzd1*^(-/-)^ dams died within 72 h of parturition and it was observed that they had insufficient milk in their stomachs. Almost all pups survived and grew normally when they were transferred to a foster dam immediately after birth. These results indicated that the *Cuzd1*^(-/-)^ dams fail to produce an adequate amount of milk.

**Fig 2:**
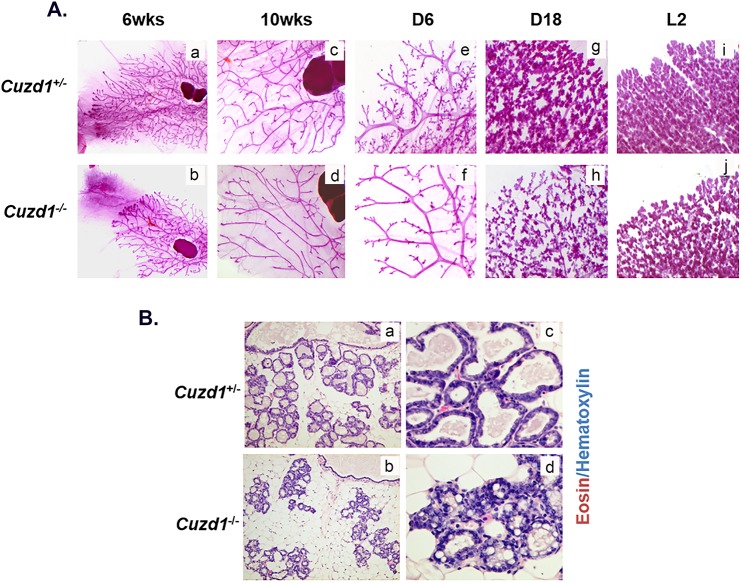
Phenotypic analysis of *Cuzd1*^(-/-)^ mice. **(A) Analysis of *Cuzd1***^**(-/-)**^ **mammary gland morphology.** Whole mount analysis of no. 4 inguinal mammary glands of virgin *Cuzd1*^(+/-)^ and *Cuzd1*^(-/-)^ mice at 6 weeks of age (a and b) and 10 weeks of age (c and d), pregnancy day 6 (e and f), pregnancy day 18 (g and h) and lactation day 2 (i and j). Magnification 4x. **(B) Histological analysis of *Cuzd1***^**(-/-)**^ **mammary gland during lactation.** Mammary gland sections of lactating (day 2) *Cuzd1*^(+/-)^ (a and c) or *Cuzd1*^(-/-)^ (b and d) mice were subjected to H&E analysis. Magnification 20x (a and b) and 40x (c and d).

To further examine the phenotypic defects in the *Cuzd1*^(-/-)^ mice, morphological analyses of whole mounts of mammary glands were performed at different stages of development. In comparison to their *Cuzd1*^(+/-)^ littermates, the expansion of the epithelial tree in *Cuzd1*^(-/-)^ mice was delayed at puberty (6-weeks old) (Fig 2A, a and b). However, smooth muscle actin (SMA) and E-cadherin staining of *Cuzd1*^(-/-)^ mammary glands at puberty indicate that there are no structural abnormalities in the cap or body cells of the terminal end buds (S2A Fig, a-d) [28–29]. The extent of ductal branching was modestly reduced in adult mutant females at estrous stage (10-weeks old) (Fig 2A, c and d). During early pregnancy, mammary glands of mutant mice exhibited a severe deficiency in tertiary branching (Fig 2A, e and f) and impaired alveolar development during late pregnancy (Fig 2A, g and h) and lactation (Fig 2A, i and j). Histological analysis of lactating *Cuzd1*^(-/-)^ mammary glands revealed sparsely distributed alveolar units with disrupted epithelial structure in comparison to their *Cuzd1*^(+/-)^ littermates (Fig 2B, a-d). Collectively, these results indicated that the impairment in alveolar differentiation in *Cuzd1*^(-/-)^ females during pregnancy and lactation leads to the deficiency in milk production.

### Loss of *Cuzd1* impairs the ErbB signaling pathway in the mammary epithelium

**Fig 3:**
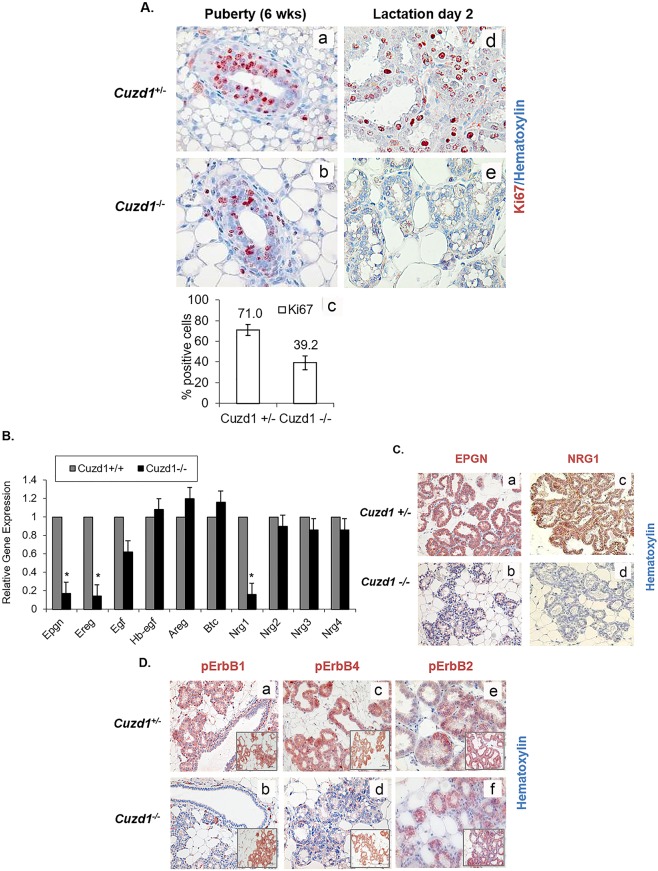
Developmental defects in *Cuzd1*^(-/-)^ mammary glands are due to an impairment in EGF signaling. **(A) Analysis of mammary epithelial cell proliferation during development.** Mammary gland sections of pubertal (6 weeks, a and b) and lactating (day 2, d and e) *Cuzd1*^(+/-)^ or *Cuzd1*^(-/-)^ mice were subjected to IHC analysis using an antibody against Ki67. Magnification 20x. The number of Ki67-positive cells during puberty in *Cuzd1*^(-/-)^ mammary tissue (a) was estimated by ImageJ software and compared with those in *Cuzd1*^(+/-)^ tissue (b). Data (c) expressed as average ± SEM of ≥3 biological replicates. **(B) Expression of EGF family ligands in the *Cuzd1***^**(-/-)**^ **mammary gland.** Total RNA was isolated from purified mammary epithelial cells of *Cuzd1*^(-/-)^ mice and *Cuzd1*^(+/-)^ littermates at day 18 of pregnancy. qPCR was performed to analyze expression levels of *Epgn*, *Ereg*, *Egf*, *Hbegf*, *Areg*, *Btc*, *Nrg1*, *Nrg2*, *Nrg3* and *Nrg4* mRNAs. Data are represented as relative gene expression ± SEM from ≥3 biological replicates. **C. Expression of EPGN and NRG1 proteins.** Mammary tissue sections obtained from *Cuzd1*^(+/-)^ and *Cuzd1*^(-/-)^ mice on lactation day 2 were subjected to IHC using antibodies specific for EPGN (a and b), NRG1 (c and d). Magnification 10x. **(D) Activation of ErbB receptors in the *Cuzd1***^**(-/-)**^ **mammary gland.** Mammary tissue sections obtained from *Cuzd1*^(+/-)^ and *Cuzd1*^(-/-)^ mice on day 18 of pregnancy were subjected to IHC using antibodies specific for pErbB1 (a and b), pErbB4 (c and d) and pErbB2 (e and f). Insets show total ErbB levels. Magnification 10x.

The impaired alveolar development in *Cuzd1*^(-/-)^ mammary glands raised the possibility that CUZD1 is involved in the control of epithelial cell proliferation. To test this possibility, we monitored the mammary epithelial proliferation in *Cuzd1*^(-/-)^ mice and *Cuzd1*^(+/-)^ littermates during puberty and lactation. We employed IHC analysis using an antibody against Ki67, a widely used marker for cellular proliferation. As expected, extensive cell proliferation was observed in the mammary ductal epithelia of non-pregnant pubertal *Cuzd1*^(+/-)^ mice (Fig 3A, a). There was a significant reduction in the number of proliferating ductal epithelial cells in the mammary glands of pubertal *Cuzd1*^(-/-)^ mice (Fig 3A, b). The difference in Ki67 positive cells at puberty is quantified in Fig 3A, c. When epithelial proliferation was assessed in the lactating mammary gland, we again observed a dramatic decline in epithelial proliferation in *Cuzd1*^(-/-)^ mice (Fig 3A, d and e). During puberty and pregnancy, *Cuzd1*^(-/-)^ females maintained normal serum levels of E, P and PRL (S2B Fig), indicating that tissue intrinsic factors rather than systemic hormonal disruptions caused by the loss of *Cuzd1* are responsible for this defect in mammary gland proliferation. These results demonstrated that *Cuzd1* plays a critical role in regulating side-branching and alveolar morphogenesis in female mice during pregnancy and lactation, in part by influencing pathways involved in mammary epithelial proliferation.

To identify the pathways downstream of CUZD1, a microarray analysis was performed to compare the gene expression profiles of mammary epithelial cells isolated from *Cuzd1*^(-/-)^ mice and their *Cuzd1*^(+/-)^ littermates on day 18 of pregnancy. This microarray identified 411 transcripts that were altered (>2-fold) in the *Cuzd1*^(-/-)^ epithelium compared to the *Cuzd1*^(+/-)^ epithelium (GEO Accession GSE30939). Prominent among the 377 down-regulated transcripts were mRNAs encoding three members of the EGF family, neuregulin-1 (*Nrg1*), epiregulin (*Ereg*) and epigen (*Epgn*). Interestingly, no significant alteration was detected in the expression levels of transcripts of several other EGF-family growth factors, such as amphiregulin (*Areg*), epidermal growth factor (*Egf*), heparin binding epidermal growth factor (*Hbegf*), neurgulin-2 (*Nrg2*), neuregulin-3 (*Nrg3*) and neuregulin-4 (*Nrg4*). Gene expression changes of EGF family ligands were confirmed using real-time RT-PCR and analyzed for statistical significance (Fig 3B). Furthermore, IHC analysis of EPGN and NRG1 at lactation day 2 showed a substantial decline in these EGF ligands in *Cuzd1*^(-/-)^ mice (Fig 3C, b and d). These data indicate that the deletion of *Cuzd1* results in reduced expression of a specific subset of EGF family ligands in the mammary epithelium during late pregnancy.

Binding of EGF ligands to ErbB receptors results in their activation via auto-phosphorylation of critical tyrosine residues, which subsequently serve as docking sites for downstream signaling molecules [30]. While EREG binds to both ErbB1 and ErbB4, EPGN acts primarily via ErbB1. NRG1 binds to ErbB3 as well as ErbB4. We therefore, examined whether the observed alterations in the expression levels of *Nrg1*, *Ereg* and *Epgn* in the mammary tissue affected the ErbB receptor-mediated signaling. Mammary gland sections obtained from mice during late pregnancy were subjected to IHC, using antibodies directed against specific phosphorylated tyrosine residues critical for activation of ErbB1 (Tyr 1068), ErbB2 (Tyr 877), and ErbB4 (Tyr 1056). Abundant activating phosphorylation of ErbB1, ErbB2, and ErbB4 was observed in mammary epithelia of *Cuzd1*^(+/-)^ mice, consistent with the proliferative activity seen in this tissue (Fig 3D, a, c, and e). In contrast, pErbB1 and pErbB4 were markedly reduced in the *Cuzd1*^(-/-)^ epithelium (Fig 3D, b and d). Interestingly, phosphorylation of ErbB2 was not affected in the *Cuzd1*^(-/-)^ epithelium (Fig 3D, f). No alteration was observed in the total protein levels of ErbB1, ErbB2, and ErbB4 in mammary epithelia of these mice (Fig 3D, insets). Collectively, these results indicated that CUZD1 is necessary for the production of the EGF family ligands, NRG1, EREG, and EPGN, which then function through ErbB receptor-mediated signaling pathways to control epithelial proliferation in the mammary gland during alveolar development.

### *Cuzd1* controls the proliferation of mammary epithelial cells by modulating the ErbB signaling pathway

**Fig 4:**
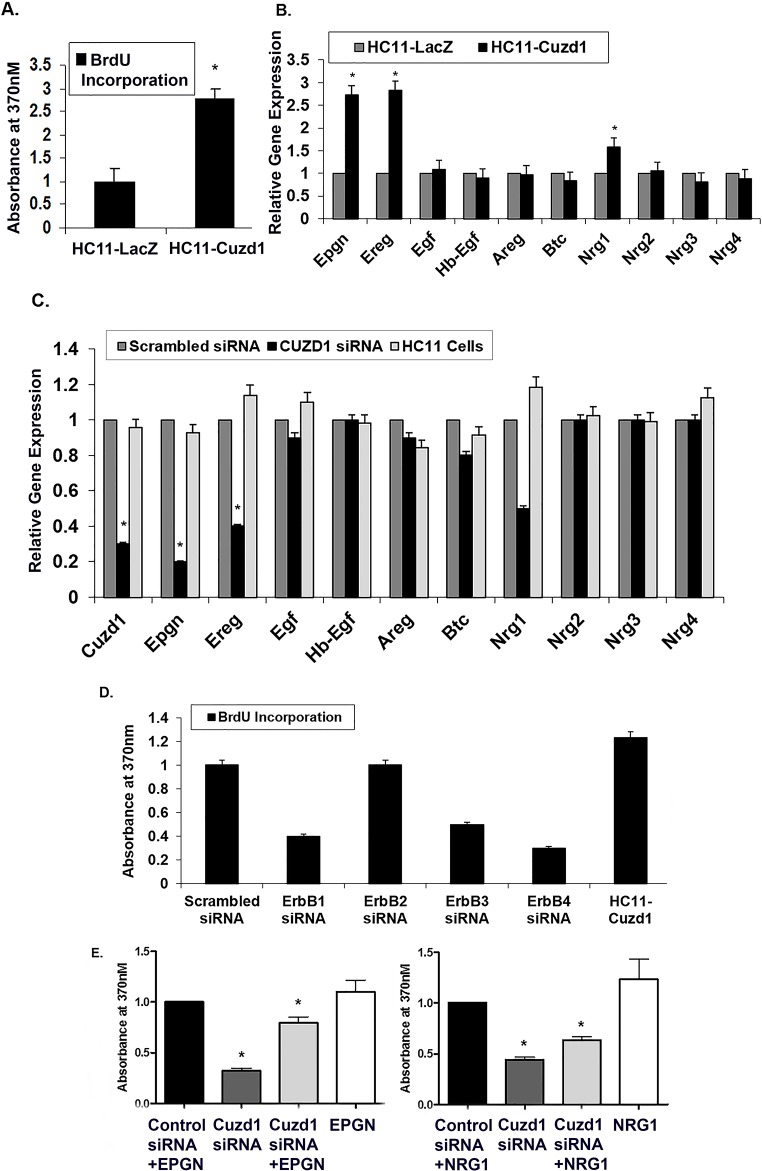
*Cuzd1* controls the expression of a subset of EGF family ligands in mammary epithelial cells. **(A) *Cuzd1* overexpressing cells exhibit increased proliferation.** HC11-Cuzd1 and HC11-LacZ cells were cultured under serum-free conditions for 48 h and 10% FBS was added along with BrdU 24 h prior to cell harvest. BrdU incorporation was measured using an ELISA-based assay. Data are expressed as Absorbance at 370nm ± SEM from ≥3 biological replicates. (**B) Expression of EGF family ligands in HC11-Cuzd1 and HC11-LacZ cells.** HC11-Cuzd1 and HC11-LacZ cells were cultured for 48 h. qPCR was performed to analyze relative expression levels of *Epgn*, *Ereg*, *Egf*, *Hbegf*, *Areg*, *Btc*, *Nrg1*, *Nrg2*, *Nrg3* and *Nrg4* mRNAs. Data are represented as relative gene expression ± SEM from ≥3 biological replicates. **(C) Expression of EGF family ligands in *Cuzd1-silenced* HC11 cells.** HC11 cells were transfected with siRNA (100nM) targeted against *Cuzd1* or scrambled siRNA (control). Total RNA was prepared from HC11 cells 48 h after transfection and subjected to qPCR using gene-specific primers to assess the expression of *Epgn*, *Ereg*, *Egf*, *Hbegf*, *Areg*, *Btc*, *Nrg1*, *Nrg2*, *Nrg3* and *Nrg4* mRNAs. Data are represented as relative gene expression ± SEM from ≥3 biological replicates. **(D) Proliferation of HC11-Cuzd1 cells upon ErbB perturbation.** HC11-Cuzd1 cells were transfected with siRNA (50nM) targeted against *ErbB1*, *ErbB2*, *ErbB3*, *ErbB4* or non-targeting siRNA (control). 48 h post transfection, the siRNA transfection mixture was removed and replaced with fresh growth medium and BrdU was administered 24 h prior to cell harvest. BrdU incorporation was measured using an ELISA-based assay. Data are expressed as Absorbance at 370nm ± SEM from ≥3 biological replicates. **(E) Proliferation of HC11 cells with *Cuzd1* knockdown and ligand supplementation.** HC11 cells were transfected with siRNA (100nM) targeted against *Cuzd1* or a non-targeting siRNA (control). 48h post-transfection, HC11 cells were supplemented with EPGN, NRG1, or a vehicle control and BrdU was added. BrdU incorporation was measured after 24h using an ELISA-based BrdU assay and resulting color reaction was measured using a plate reader at 370nm. Data are expressed as Absorbance at 370nm ± SEM from ≥3 biological replicates.

We used HC11 cells, a non-transformed mammary epithelial cell line derived from pregnant mice, to examine the cell autonomous role of *Cuzd1* [31]. A lentiviral expression vector harboring a full-length cDNA encoding *Cuzd1* or *LacZ* (control) was integrated into HC11 cells to generate stable cell lines which express constitutively elevated levels of *Cuzd1* (HC11-Cuzd1) or *β-galactosidase* (HC11-LacZ) (S3A and S3B Fig). When HC11-Cuzd1 cells were subjected to a BrdU incorporation assay, they exhibited significantly higher rates of proliferation compared to control HC11-LacZ cells (Fig 4A). These data provided evidence that *Cuzd1*-dependent mechanisms indeed promote proliferation of mammary epithelial cells.

We next investigated whether CUZD1 controls the proliferation of HC11 cells by regulating the expression of the EGF growth factors. First, we examined the effects of *Cuzd1* overexpression on the expression of the EGF family members. Significantly higher levels of *Epgn*, *Ereg* and *Nrg1* transcripts were detected in HC11-Cuzd1 cells as compared to the HC11-LacZ cells (Fig 4B). Conversely, siRNA-mediated attenuation of *Cuzd1* mRNA expression in HC11 cells led to a marked reduction in the levels of *Nrg1*, *Ereg*, and *Epgn* mRNAs without significantly altering the levels of mRNAs encoding other EGF family ligands (Fig 4C). To determine which ErbB receptors play a role in CUZD1-induced cell proliferation, we performed a knock down of ErbB receptors 1–4 in HC11-Cuzd1 cells using gene-specific siRNAs (S4 Fig). Knock down of ErbB1, ErbB3 and ErBb4 resulted in a decrease in HC11-Cuzd1 cell proliferation as measured by a BrdU incorporation assay (Fig 4D). We next wanted to determine if the loss of *Cuzd1*, and therefore the loss of specific EGF family ligands, led to a reduction in mammary epithelial cell proliferation. Using siRNA, we knocked down *Cuzd1* in HC11 mammary epithelial cells and supplemented with EPGN or NRG1 ligands. EPGN and NRG1 were both able to partially rescue proliferation of HC11 cells as compared to the ligand treated control (Fig 4E). Altogether, these data strongly support the concept that CUZD1 controls the production of specific EGF family growth factors, which act via ErbB1, ErbB3 and ErbB4 to induce mammary epithelial cell proliferation.

### CUZD1-mediated STAT5 signaling is necessary for differentiation of the mammary gland

To further elucidate the molecular mechanism of CUZD1, we attempted to identify the cellular factors that interact with it. To achieve this goal, we created HC11 cells stably over-expressing recombinant FLAG epitope-tagged CUZD1 (HC11-3xFLAG-Cuzd1 cells). Soluble extracts of these cells were subjected to co-immunoprecipitation using a FLAG antibody. The immunoprecipitated proteins were recovered and submitted for mass spectrometry. The LC/MS identified peptide fragments corresponding to multiple potential interaction partners of CUZD1, including JAK1 and JAK2, protein arginine methyltransferase 5 and phosphoribosyl pyrophosphate synthetase 1.

**Fig 5:**
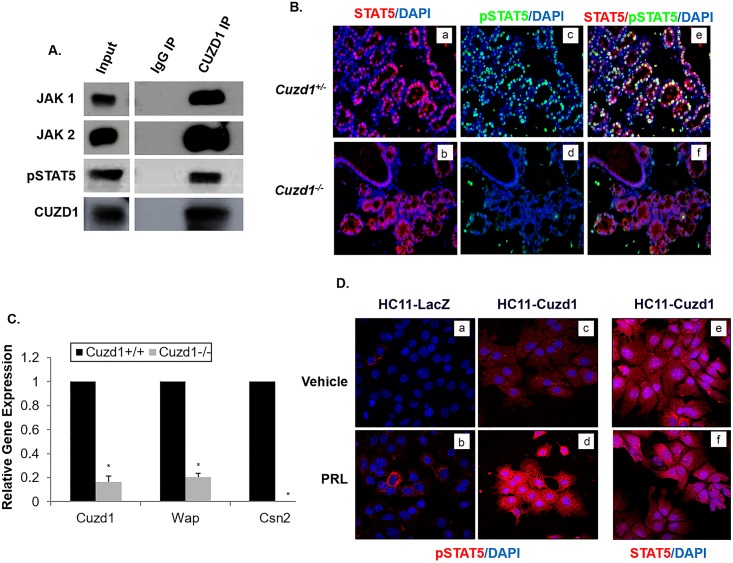
CUZD1-mediated STAT5 signaling is necessary for PRL-induced proliferation and differentiation of the mammary gland. **(A) CUZD1 associates with JAK1/2 and STAT5.** HC11 cells were cultured for 48 h in serum-free media and then exposed to 10% FBS for 24 h. Cells were lysed and samples were immunoprecipitated with an IgG (control) or CUZD1 antibody. CUZD1 and the associated proteins were confirmed by Western blot analysis. Blots were probed with CUZD1, JAK1, JAK2 and pSTAT5 antibodies. **(B) Activation STAT5 in the *Cuzd1***^**(-/-)**^ **mammary gland.** Mammary tissue sections obtained from *Cuzd1*^(+/-)^ and *Cuzd1*^(-/-)^ mice on day 18 of pregnancy were subjected to IHC using an antibody specific for pSTAT5 and total STAT5. Magnification 10x. **(C) Expression of milk protein genes in the *Cuzd1***^**(-/-)**^ **mammary gland.** RNA was isolated from mammary glands of lactating day 2 mammary glands from *Cuzd1*^(+/-)^ and *Cuzd1*^(-/-)^ mice and analyzed using primers specific for *Cuzd1*, *Wap*, and *Csn2*. Data are represented as relative gene expression ± SEM from ≥3 biological replicates. **(D) Analysis of pSTAT5 in HC11-LacZ and HC11-Cuzd1 cells.** HC11-LacZ and HC11-Cuzd1 cells were cultured for 48 h in serum-free media and then exposed to a vehicle control (a, c, and e) or PRL (b, d, and f) for 24 h. Cells were fixed and subjected to ICC using an antibody specific for pSTAT5 (a, b, c, and d) or total STAT5 (e and f). Magnification 40x.

Since JAK1/2 signaling and subsequent STAT5 phosphorylation is critical for mammary gland development, we focused on the interactions between JAK1, JAK2, and CUZD1. Co-immunoprecipitation of JAK1 and JAK2 from HC11 cell lysates was confirmed using an IP for endogenous CUZD1 and Western blot analysis (Fig 5A). Interestingly, we also detected a signal for phosphorylated STAT5 in the HC11 cell immunoprecipitates (Fig 5A). The presence of this complex of proteins was also confirmed using Western blot in HC11-3xFLAG-Cuzd1 cells (S5A Fig). Although STAT5 was not identified as an interacting partner of CUZD1 in our proteomic analysis of the immunoprecipitate, it is conceivable that CUZD1 interacts directly with JAK1/JAK2, which exist in a larger cytosolic complex with STAT5.

In response to signaling by hormones, such as prolactin, activation of JAK1/2 leads to activation of the transcription factors STAT5a and STAT5b, which control mammary epithelial cell proliferation and differentiation during alveologenesis [7,32]. Though both STAT5a and STAT5b are present in the mammary gland, STAT5a is the dominant form phosphorylated and localized to the nucleus during pregnancy and lactation [18,33]. We examined the status of the activating STAT5 phosphorylation (Tyr 694) in the mammary glands of *Cuzd1*^(-/-)^ mice at day 18 of pregnancy by IHC analysis. Total STAT5 protein levels were unchanged in *Cuzd1*^(-/-)^ mice compared to *Cuzd1*^(+/-)^ (Fig 5B, a and b). However, we observed a striking loss of STAT5 phosphorylation in the mammary epithelia of these mice, whereas abundant pSTAT5 was present in the mammary epithelium of Cuzd1^(+/-)^ littermates (Fig 5B, c and d, e and f). STAT5 is known to directly regulate the expression of *Wap* and *Csn2*, two milk proteins secreted by differentiated epithelial cells [8,19]. We postulated that the loss of STAT5 phosphorylation impairs STAT5-dependent gene expression, leading to the observed deficiency in milk production in *Cuzd1*^(-/-)^ females. To test this notion, we analyzed the gene expression levels of *Wap* and *Csn2*. The levels of *Wap* and *Csn2* transcripts were indeed markedly reduced in the mammary glands of *Cuzd1*^(-/-)^ females during lactation (Fig 5C). These results formed the basis of our hypothesis that CUZD1-mediated signaling through JAK/STAT5 controls mammary epithelial cell differentiation.

To further understand the functional significance of the interaction of CUZD1 with the JAK/STAT5 pathway, we examined phosphorylation and localization of STAT5 and expression of direct transcriptional targets of pSTAT5 in HC11-Cuzd1 cells in response to PRL treatment. In this experiment, the HC11-Cuzd1 cells were treated with vehicle or PRL and STAT5 phosphorylation/localization was analyzed using immunocytochemistry. We observed that pSTAT5 immunostaining was dramatically enhanced in HC11-Cuzd1 cells (Fig 5D, c and d) relative to HC11-LacZ (Fig 5D, a and b) cells upon PRL treatment and, as expected, it was localized predominantly in the nucleus. PRL treatment of HC11-Cuzd1 cells did not result in a marked alteration in total STAT5 levels (Fig 5D, e and f). The enhanced STAT5 phosphorylation observed in HC11-Cuzd1 cells as compared to HC11-LacZ cells was also confirmed via Western blotting (S5B Fig). These data are consistent with the concept that CUZD1 promotes PRL signaling by enhancing STAT5 phosphorylation and activation.

### Loss of *Cuzd1* impairs prolactin-induced lobuloalveologenesis

**Fig 6:**
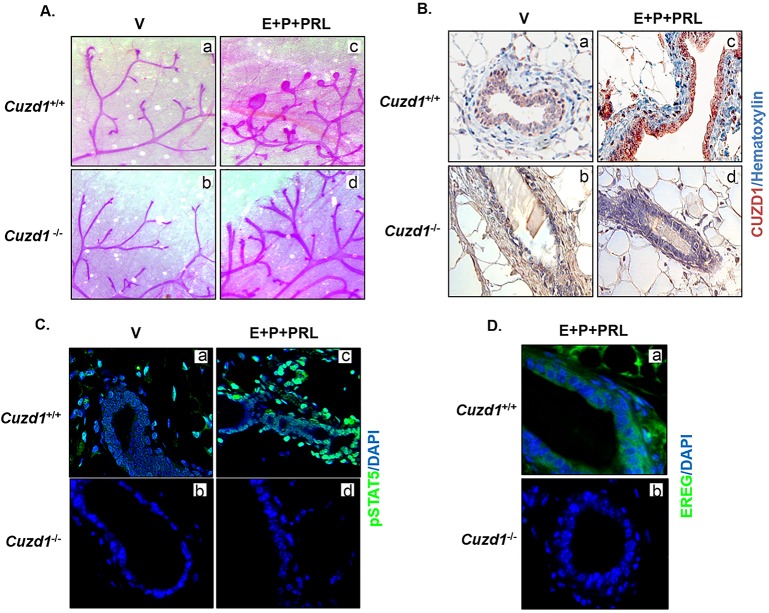
*Cuzd1*^(-/-)^ mammary glands do not undergo alveologenesis in response to hormone treatment. **(A) *Cuzd1***^**(-/-)**^ **mammary gland morphology in response to hormonal treatment.** *Cuzd1*^(+/+)^ and *Cuzd1*^(-/-)^ mice (n = 5) were treated with a vehicle control (a and b) or E+P+PRL (c and d) for 3d. Whole mount analysis of no. 4 inguinal mammary glands of *Cuzd1*^(+/+)^ and *Cuzd1*^(-/-)^ mice after 3d of hormone treatment. Magnification 6.3x. **(B) Analysis of CUZD1 expression.** Mammary tissue sections obtained from *Cuzd1*^(+/+)^ (a and c) and *Cuzd1*^(-/-)^ (b and d) mice were subjected to IHC using an antibody specific for CUZD1. **(C) Activation of STAT5.** Mammary tissue sections obtained from *Cuzd1*^(+/+)^ (a and c) and *Cuzd1*^(-/-)^ (b and d) mice were subjected to IHC using an antibody specific for pSTAT5. **(D) Analysis of EREG expression.** Mammary tissue sections obtained from *Cuzd1*^(+/+)^ (a) and *Cuzd1*^(-/-)^ (b) mice were subjected to IHC using an antibody specific for EREG. Magnification 20x.

To investigate the role of CUZD1 in the PRL signaling pathway *in vivo*, virgin, pubertal *Cuzd1*^(-/-)^ and *Cuzd1*^(+/+)^ (wild type) mice were treated with E, P and PRL for 3 consecutive days to stimulate proliferation and differentiation of the mammary epithelium. To examine the gross morphological changes in the mammary epithelium following this hormonal treatment, whole mounts of mammary glands were performed. Compared to the vehicle control, *Cuzd1*^(+/+)^ mice treated with E, P and PRL exhibited initiation of alveolar development (Fig 6A, a and c). Conversely, *Cuzd1*^(-/-)^ mice displayed a markedly reduced response to E, P and PRL treatment compared to vehicle control (Fig 6A, b and d). Interestingly, we observed an elevated CUZD1 expression and nuclear localization in the mammary epithelium of *Cuzd1*^(+/+)^ mice treated with E, P and PRL compared to vehicle-treated controls (Fig 6B, a and c). As expected, this induction was absent in *Cuzd1*^(-/-)^ mice (Fig 6B, b and d).

We also observed a robust phosphorylation of STAT5 and its nuclear localization in mammary epithelia of mice treated with E, P and PRL, which was absent in *Cuzd1*^(-/-)^ mice (Fig 6C, a-d). Consistent with data obtained in cell lines, the expression of EREG, a direct target of STAT5, which is induced in wild-type mice upon treatment with E, P, and PRL, was absent in *Cuzd1*^(-/-)^ mice (Fig 6D, a and b). Overall, these data support the concept that CUZD1 is necessary for transduction of PRL signaling through the JAK/STAT pathway to induce mammary epithelial gene expression during hormone-induced alveologenesis.

### CUZD1 and STAT5 co-occupy regulatory regions of target genes

**Fig 7:**
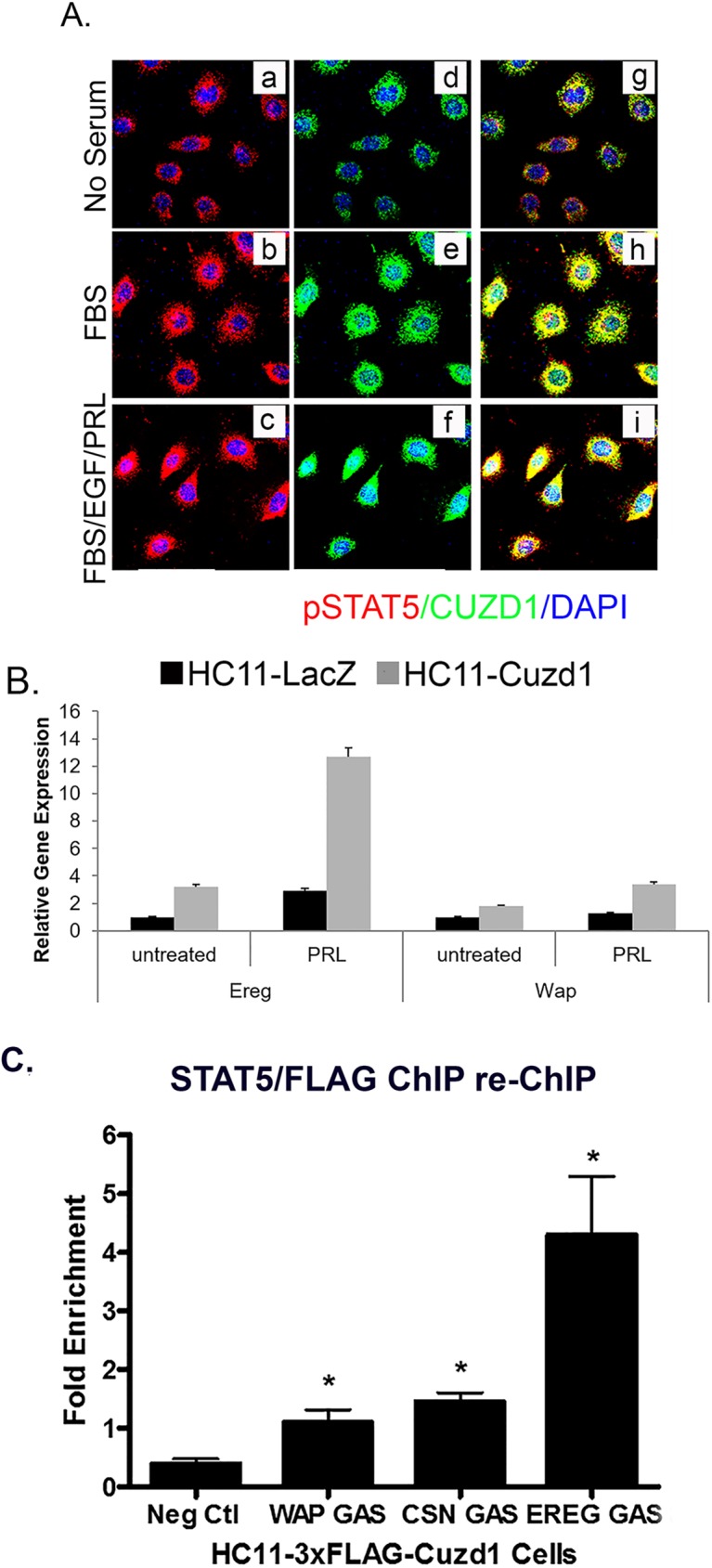
CUZD1 and STAT5 translocate to the nucleus and modulate target gene expression. **(A) CUZD1 translocates to the nucleus in response to culture serum.** HC11-Cuzd1 cells were cultured for 48 h in serum-free media and followed by media supplemented with FBS only or a cocktail of FBS/PRL/EGF for additional 24 h. Cells were subjected to ICC using an antibody specific for CUZD1 and pSTAT5. Magnification 40X. **(B) Transcription of STAT5 target genes is activated in response to PRL.** HC11-Cuzd1 cells were cultured for 48 h in serum-free media and treated with PRL for 24 h. Cells were subjected to qPCR to assess the relative levels of mRNA expression for STAT5 regulated-target genes, *Ereg* and *Wap*. Data are represented as relative gene expression ± SEM from ≥3 biological replicates. **(C) STAT5 remains in a complex with CUZD1 when bound to DNA regulatory elements.** HC11-3xFLAG-Cuzd1 cells were cultured with FBS, EGF and PRL for 6h. Protein/DNA complexes were precipitated using an antibody for STAT5 followed by FLAG (anti-M2), and subjected to qPCR using primers to GAS motifs of *Wap*, *Csn2*, and *Ereg* promoters, respectively. Data are represented as fold enrichment ± SEM from ≥3 biological replicates.

**Fig 8:**
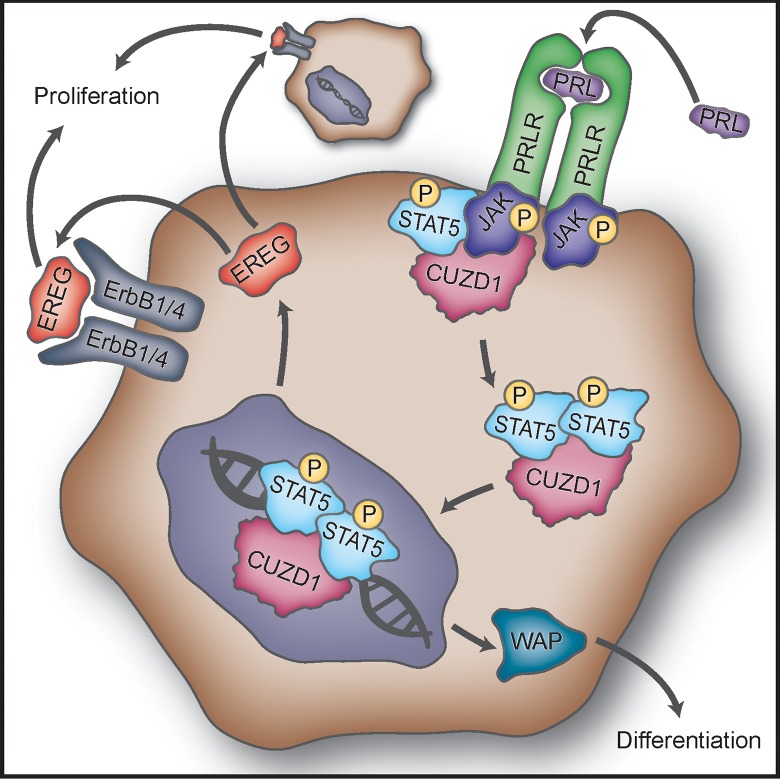
Proposed mechanism of action of CUZD1 in the mammary gland epithelium. Binding of PRL to PRLR induces activation of JAK1/2. CUZD1 forms a complex with JAKs and potentiates activation of STAT5 downstream of PRLR. Activated STAT5 and CUZD1 translocate to the nucleus where STAT5 regulates transcription of target genes, such as *Ereg* and *Wap*. EREG acts in a paracrine and/or autocrine manner through ErbB1 and/or ErbB4 to induce mammary epithelial proliferation. The expression of WAP, a milk protein, marks the terminal differentiation of the mammary epithelium.

CUZD1 has no nuclear localization sequence or DNA binding domain, but we observed that it was translocated to the nucleus upon stimulation with serum. We hypothesized that CUZD1 could be moving into the nucleus in association with pSTAT5. To investigate this possibility, HC11-3xFLAG-Cuzd1 cells were treated with a PRL/FBS/EGF cocktail, FBS, or a vehicle control to induce nuclear translocation of pSTAT5. Dual immunostaining was then performed to examine the cellular locations of pSTAT5 and CUZD1. In cells treated with the vehicle control, pSTAT5 and CUZD1 remained largely cytoplasmic (Fig 7A, a, d, and g). Upon stimulation with FBS or PRL/FBS/EGF, pSTAT5 and CUZD1 were colocalized in the nucleus (Fig 7A, b-c, e-f, and h-i). Previous studies reported that STAT5 binds directly to regulatory regions of *Ereg* and *Wap* genes to regulate their transcription (15,16). We observed that the expression of *Ereg* and *Wap* genes were up-regulated upon treatment with PRL, and their expression was further elevated in *Cuzd1*-overexpressing HC11 cells (Fig 7B). Based on the protein structure of CUZD1 (S1A Fig), there is no indication that CUZD1 binds to DNA, but we wanted to determine if CUZD1 and STAT5 remained in a complex when STAT5 is bound to DNA. To investigate this, we performed a ChIP re-ChIP using a STAT5-specific antibody followed by precipitation with anti-FLAG (M2) resin. Enrichment of regulatory elements in specific GAS motifs of *Ereg*, *Wap*, and *Csn2* indicated that CUZD1 remains bound to STAT5 in the nucleus when STAT5 is acting as a transcription factor (Fig 7C). In single ChIP experiments, we confirmed STAT5 binding at in the *Ereg*, *Wap*, and *Csn2* GAS sequences (S6 Fig) as well as enrichment of these regulatory elements when we immunoprecipitated the FLAG-CUZD1 fusion protein (S6 Fig). The authenticity of this result was confirmed by the absence of enrichment of the *Wap*, *Csn2* and *Ereg* GAS sites when a FLAG ChIP was performed in HC11-Cuzd1 cells in which CUZD1 is not flag-tagged (S6 Fig). Collectively, our results indicated that, upon PRL-induced activation, the CUZD1-STAT5 complex translocates to the nucleus and interacts with target genes to bring about changes in gene expression that critically promote mammary epithelial cell proliferation and differentiation (Fig 8).

## Discussion

E, P and PRL act in concert with the EGF family growth factors to govern mammary gland development during pregnancy and lactation [3,13]. In this study, we provide evidence that CUZD1 is a novel regulator of STAT5 signaling in the steroid-primed mammary epithelium. Loss of *Cuzd1* expression in mammary epithelial cells prevented *in vivo* phosphorylation of STAT5, resulting in a severe impairment in mammary epithelial proliferation and differentiation, which disrupts alveologenesis and prevents milk production during lactation.

A molecular link between CUZD1 and STAT5 phosphorylation has emerged from our study. Immunoprecipitation of CUZD1 from mammary epithelial cells followed by mass spectrometric and Western blot analyses revealed that CUZD1 is physically associated with several proteins, including JAK1/JAK2 and STAT5. Importantly, increased CUZD1 expression augmented PRL-induced phosphorylation as well as nuclear translocation of STAT5. The precise nature of CUZD1’s association with the JAK1/JAK2/STAT5 complex and the mechanism by which it promotes STAT5 phosphorylation are presently unclear. It is conceivable that CUZD1 potentiates JAK/STAT signaling downstream of PRLR activation by acting as an adaptor protein that aids in the recruitment of STAT5 to the PRLR/JAK complex. It may also act in an accessory role in stabilizing/enhancing phosphorylation of STAT5 by JAKs. Precedence for this hypothesis is based on literature describing the roles of effector proteins that alter signaling through this complex [34]. For example, c-Src has been shown to propagate PRL initiated JAK/STAT signaling in normal mammary tissue [35]. Additionally, caveolin-1 (Cav-1) has been shown to inhibit the STAT5 signaling pathway by competitively binding to the tyrosine kinase domain of JAK2, preventing interaction and subsequent activation of STAT5 [36].

Female mice lacking *Prlr*, *Jak2*, and *Stat5* are characterized by severe defects in mammary lobuloalveologenesis during pregnancy and lack of milk production during lactation [14,20,33,37–39]. The *Cuzd1*^(-/-)^ mice phenocopy the mammary defects observed in these mice during pregnancy and lactation, lending further support to the concept that CUZD1 is functionally linked to the components of the PRLR/JAK/STAT5 pathway during lobuloalveolar development and lactation.

The CUZD1 protein is localized in both cytoplasmic and nuclear compartments of mammary epithelial cells. When the mammary epithelial cells are grown in the absence of serum, CUZD1 is predominantly localized in the cytoplasm. Stimulation of these cells with media containing serum triggers nuclear translocation of CUZD1. This result is also recapitulated by adding a combination of PRL, EGF and serum to these cells. Since CUZD1 lacks a nuclear localization motif or a DNA binding domain, we predicted that its translocation to the nucleus is dependent on association with a transcription factor. Indeed, our results are consistent with the view that CUZD1 translocates to the nucleus in association with pSTAT5. We further demonstrated that CUZD1 is recruited along with pSTAT5 to the regulatory regions of key target genes, such as *Ereg* and *Wap*. It is plausible that EREG contributes to CUZD1-mediated epithelial proliferation and alveolar expansion during pregnancy and lactation, as *Ereg* is a direct transcriptional target of STAT5 and has been implicated in promoting growth and survival of breast cancer cells [40–42].

Our study showed that CUZD1 controls the production of a subset of EGF family growth factors, EREG, NRG1, and EPGN, in mammary epithelium during pregnancy. Mice lacking *Nrg1* display pronounced defects in mammary alveologenesis with condensed alveoli and impaired alveolar outgrowth during pregnancy [43,44]. Development of mutant mouse models showed that ErbB1, ErbB2, and ErbB3 play important roles in mammary ductal growth and fat pad penetration [45–48]. *ErbB4*^(-/-)^ mammary glands exhibited severe defects in alveolar proliferation and differentiation during pregnancy and lactation [21]. *Cuzd1*^(-/-)^ mammary glands showed impaired activation of ErbB1 and ErbB4 during pregnancy and lactation. These results are in accord with the hypothesis that the CUZD1-regulated growth factors, NRG1, EREG and EPGN, act primarily through ErbB1 and ErbB4 to exert their effects mainly during alveolar development. Consistent with this concept, there is a remarkable similarity between the mammary gland phenotypes of *ErbB4*^(-/-)^ and *Cuzd1*^(-/-)^ females.

In summary, our findings support a model in which CUZD1 is a downstream mediator of PRL that enhances the signaling pathway through STAT5 during proliferation and differentiation of the mammary epithelium (Fig 8). CUZD1 impacts mammary epithelial proliferation and differentiation during pregnancy and lactation. It promotes production of a specific subset of the EGF-like ligands, NRG1, EREG and EPGN, which control alveolar development. These growth factors primarily function through ErbB1 and ErbB4 to regulate the proliferation and differentiation of mammary epithelial cells. Further analysis of the molecular mechanisms by which CUZD1 integrates the pathways regulated by STAT5 and the EGF family growth factors will improve our understanding of the molecular networks that underlie PRL regulation of normal mammary gland development.

## Materials and methods

### Animals

Mice were maintained in the designated animal care facility at the University of Illinois, according to institutional guidelines for the care and use of laboratory animals. All experimental procedures involving mice were conducted in accordance with National Institutes of Health standards for the use and care of mice. The animal protocol describing these procedures was approved by the University of Illinois Institutional Animal Care and Use Committee (IACUC). The IACUC approval number for this protocol is 16026. This approval is valid until August 22, 2019.

### Gene targeting

To generate the vector for homologous recombination, about 16-kb mouse genomic DNA containing eight exons of mouse *Cuzd1* was sequenced and intron-exon boundaries were analyzed. A 4.0-kb *BamH I-Kpn I* fragment containing the 1^st^ and 2^nd^ exons and a 2.0-kb BamH I-EcoR I fragment containing part of 6^th^ exon was cloned into Scrambler A and B site of pKO Scrambler NTKV-1901 targeting vector, respectively. Correct targeting resulted in deletion of gene sequence containing exons III-VI spanning the first and second CUB domains of CUZD1 protein and replaced with a neomycin resistance gene (NEO) (S1B Fig). The construct was linearized and electroporated into embryonic stem (ES) cells. ES clones were selected by G418 and screened by Southern blot analysis employing a 335-bp 5’-end probe, a 390-bp 3’-end probe and a 450-bp internal probe respectively. The ES clone with appropriate homologous recombination was selected for blastocyst injection and chimaeras were generated with heterozygous ES cell lines. Heterozygous male mice were backcrossed to wildtype C57BL/6 female to generate the *Cuzd1*-null mice with pure genetic background. Progeny were genotyped by using PCR assay that identified both mutant and wild-type alleles and Southern blotting analysis with 5’-end probe (S1B Fig).

### Cell line and cell culture

HC11 cells were grown in RPMI-1640 supplemented with 5% (v/v) fetal bovine serum, 1x Penicillin-Streptomycin, 10 ng/ml EGF and 5 μg/ml insulin. In certain experiments, 2% charcoal-stripped calf serum was used.

### Hormone treatment

HC11 cells were treated with 10nm E, 10μm P and/or 50μm PRL. Ovariectomized mice were treated with vehicle controls (oil or saline), 1ng E and 1mg P (subcutaneous) and/or 50μg/g bw of PRL (intraperitoneal).

### Whole mount analysis of mammary gland morphology

The inguinal mammary glands were dissected out, spread onto a glass slide and fixed in a 1:3 mixture of glacial acetic acid/100% ethanol. After hydration, slides were stained as described previously [50]. Following mounting, images were captured using bright field dissecting microscope.

### Immunochemistry

Paraffin-embedded mammary tissues were sectioned and subjected to IHC as described previously [49]. Rabbit polyclonal antibodies against a peptide antigen containing amino acids SSPNYPKPHPEL of mouse CUZD1 were generated in our laboratory. IHC was performed on mammary tissue sections, using primary antibodies and bound primary antibodies were detected with either horseradish peroxidase (HRP)- or fluorescent label-conjugated secondary antibodies. Sections were counterstained with hematoxylin or dapi and mounted.

Cells were fixed in a 3.7% formalin solution at room temperature for 15 min followed by washing with PBS. The cells were permeabilized by 0.25% Triton X-100 in PBS for 10 min, and nonspecific binding of antibodies was blocked with 5% donkey serum for 1 h at room temperature. Cells were incubated with primary antibodies overnight at 4°C. Labeling was visualized with fluorescent label-conjugated secondary antibodies and slides were mounted in Prolong GOLD and cured for 24 h before imaging. The images of immunohistochemical staining were captured by using a Leica DM2500 light microscope fitted with a Qimaging Retiga 2000R camera (Qimaging). Immunofluorescence imaging was performed on a Leica 700 confocal microscope. These images were minimally processed on ADOBE Photoshop version 8.

### Isolation of mammary epithelial cells and DNA microarray analysis

Pooled inguinal mammary glands from three mice (*Cuzd1*^(+/-)^ or *Cuzd1*^(-/-)^) were minced into small pieces and incubated with DMEM: F12 containing 100 U/ml hyaluronidase and 1.5 mg/ml collagenase at 37°C for 2 h accompanied by shaking at 110 RPM. Following neutralization of enzyme activity with 5% FBS, the homogeneous cell mixture was centrifuged and the cell pellet was washed several times with PBS. Purified epithelial cells were frozen in liquid nitrogen and stored at –80°C.

Total RNA was prepared from these cells and hybridized to Affymetrix mouse arrays (GeneChipMouse Genome 430 2.0 array) containing probes that represented ~14,000 known genes. They were processed and analyzed according to Affymetrix protocol. Although the microarray analysis was performed using pooled mammary glands, we further confirmed gene expression changes, using RNA samples isolated from independent batches of epithelial cells isolated from *Cuzd1*^(+/-)^ or *Cuzd1*^(-/-)^ glands and analyzing the expression of selected genes by real-time PCR followed by statistical analyses. As shown in Fig 3B, several transcripts corresponding to the EGF family ligands were indeed differentially expressed in a manner similar to that predicted by the microarray analysis. The microarray data were deposited in the publicly available GEO database with GEO Accession GSE30939.

### Quantitative real-time PCR (qPCR) analysis

For qPCR, total RNA was extracted from purified mammary epithelium or cultured HC11 cells using Trizol RNA purification kit, according to manufacturer’s instructions and subjected to qPCR using gene specific primers. Primer sequences are provided in S1 Table. Relative mRNA levels were plotted after normalization to the loading control 36B4. The error bars represent the relative gene expression ± the standard error from three or more independent trials. Data were analyzed using a student’s t-test and * indicate p-values < 0.05.

### siRNA treatment

HC11 cells were transfected with siRNA against *Cuzd1* or control siRNA (non-targeting), using Lipofectamine-RNAimax reagent following manufacturer’s protocol. Briefly, lipofectamine was mixed with siRNA, and allowed to form siRNA-liposome complexes, which were then added to HC11 cells at 60% confluency. After 24 h, the transfection was repeated again. Cells were harvested 48 h after the second transfection, total RNA was isolated and analyzed by qPCR using gene-specific primers.

### Immunoprecipitation

HC11-3xFLAG-Cuzd1 cells were cultured with FBS, EGF and PRL for 6h, lysed and samples were precleared before immunoprecipitation (IP). The IP was done using anti-FLAG M2 or a mouse IgG control resin (according to manufacturer’s directions) and the captured proteins were eluted using 3xFLAG peptide. Samples were boiled in SDS buffer and analyzed by standard Western blotting.

### Mass spectrometry

Directly following IP, protein samples were submitted to the Mass Spectrometry Laboratory at the University of Illinois at Urbana-Champaign. Liquid chromatography (LC)/mass spectrometry (MS) proteomic data were analyzed using Mascot (Matrix Science) and results were sorted by protein score.

### Chromatin immunoprecipitation

ChIP assays were performed using the EZ-ChIP kit (Millipore) according to the manufacturer's instructions with minor modifications. Anti-FLAG M2 affinity gel (Sigma, A2220) and anti-STAT5 antibody (Santa Cruz, sc-835) were used overnight at 4°C to immunoprecipitate flag-CUZD1 and STAT5, respectively. Normal mouse IgG (Santa Cruz, sc-2027) immunoprecipitation served as a negative control. Protein/DNA complexes were eluted, crosslinks were reversed and purified DNA was analyzed for enrichment in sequences of interest using qPCR.

## Supporting information

S1 Fig Targeting strategy for the *Cuzd1* locus. **(A) Protein structure of CUZD1.** CUZD1 contains two tandem CUB (Complement subcomponent /C1s, Uegf, Bmp1) motifs and a zona-pellucida (ZP)-like domain. **(B) Map of *Cuzd1* target**. The genomic organization of the wild-type *Cuzd1* allele is shown with black boxes representing exons and white boxes representing introns. In the targeting vector, the neomycin (NEO) resistance gene is included to provide clone selection. Homologous recombination results in replacement of exons III-VI by the NEO resistance gene. P1, P2, P3 represent the locations of primers used for genotyping PCR to identify wild-type or null genomic mutation. A subset of restriction enzyme sites is shown for relative orientation and targeting vector construction: B, BamHI; E, EcoRI; X, XhoI. **(C) Genotyping of *Cuzd1*-null mice.** Genotyping was performed by PCR using tail genomic DNA as template and P1 and P2 or P1 and P3 as primers. The 513 bp and 782 bp DNA fragments arose from wild-type and mutant loci, respectively. **(D) Measurement of *Cuzd1* mRNA.** Total RNA was isolated from pregnant (day 1) uteri of heterozygous *Cuzd1*^(+/-)^ and homozygous *Cuzd1*^(-/-)^ mice. The RNA was subjected to Northern blotting, using P^32^-labled probes specific for *Cuzd1* and internal control gene, 36B4. (+/+), (+/-), and (-/-) represent genomic DNA of wild-type, heterozygous and homozygous mice, respectively.
(TIF)

S2 Fig Phenotypic analysis of *Cuzd1*^(-/-)^ mice. **(A) Expression of SMA and E-cadherin in terminal end buds.** Mammary tissue sections obtained from *Cuzd1*^(+/-)^ and *Cuzd1*^(-/-)^ mice were subjected to IHC using an antibody specific for SMA (a and b) and E-cadherin (c and d). **(B) Hormone level measurements.** Blood samples were collected from heterozygous *Cuzd1*^(+/-)^ and homozygous *Cuzd1*^(-/-)^ mice on day 18 of pregnancy. The measurements of E, P and PRL levels were performed as described in Experimental Procedures. Bars represent average values ± SEM from five animals of each genotype.
(TIF)

S3 Fig Overexpression of Cuzd1 in HC11-Cuzd1 cells. HC11 cells were transduced with lentivirus harboring *Cuzd1* or *LacZ* cDNA to create stable cells overexpressing *Cuzd1* (HC11-Cuzd1) or *LacZ* (HC11-LacZ), respectively. HC11-LacZ and HC11-Cuzd1 cells were lysed and total protein extracts were analyzed using Western blot. Blots were probed with an antibody specific for CUZD1. Calnexin was used as a loading control (a). The density of the bands was quantified using ImageJ and are expressed as Band Intensity (b).
(TIF)

S4 Fig Confirmation of individual *ErbB* knockdown. HC11-Cuzd1 cells were transfected with siRNA (50nM) targeted against *ErbB1*, *ErbB2*, *ErbB3*, *ErbB4* or scrambled siRNA (control). Total RNA was isolated from these cells and subjected to real-time PCR using specific primers to validate *ErbB 1–4* mRNA expression. Data are represented as relative gene expression ± SEM.
(TIF)

S5 Fig **(A) Confirmation of CUZD1 protein complex in HC11-3xFLAG-Cuzd1 cells.** HC11-3xFLAG-Cuzd1 cells were cultured for 48 h in serum-free media and then treated with FBS/PRL/EGF for 6 h. Cells were lysed and samples were immunoprecipitated with an IgG (control) or M2 (anti-FLAG) antibody. 3xFLAG-CUZD1 and the associated proteins were confirmed by Western blot analysis. Blots were probed with FLAG, JAK1, JAK2 and pSTAT5 antibodies. **(B). Alteration in STAT5 phosphorylation in *Cuzd1* overexpressing cells.** HC11-LacZ and HC11-Cuzd1 cells were lysed and total protein extracts were analyzed using Western blot. Blots were probed with an antibodies specific to STAT5 and pSTAT5. Calnexin was used as a loading control (a). The density of the bands was quantified using ImageJ and are expressed as Band Intensity (b).
(TIF)

S6 Fig Confirmation ChIP with individual antibodies. HC11-3xFLAG-Cuzd1 and HC11-Cuzd1 cells were cultured with FBS/EGF/PRL for 6h. Protein/DNA complexes were precipitated using an antibody for STAT5 or FLAG, and subjected to qPCR using primers specific to GAS motifs of *Wap*, *Csn2*, and *Ereg* promoters, respectively. Data are represented as relative gene expression ± SEM.
(TIF)

S1 Text Reagents List. (DOCX)

S1 Table Primer Sequences. (DOCX)
