# Supplementary figures and images for: CUZD1 is a critical mediator of the JAK/STAT5 signaling pathway that controls mammary gland development during pregnancy
Source: PLoS Genet. 2017 Mar 9;13(3):e1006654. doi: 10.1371/journal.pgen.1006654 (PMC5363987; doi:10.1371/journal.pgen.1006654)

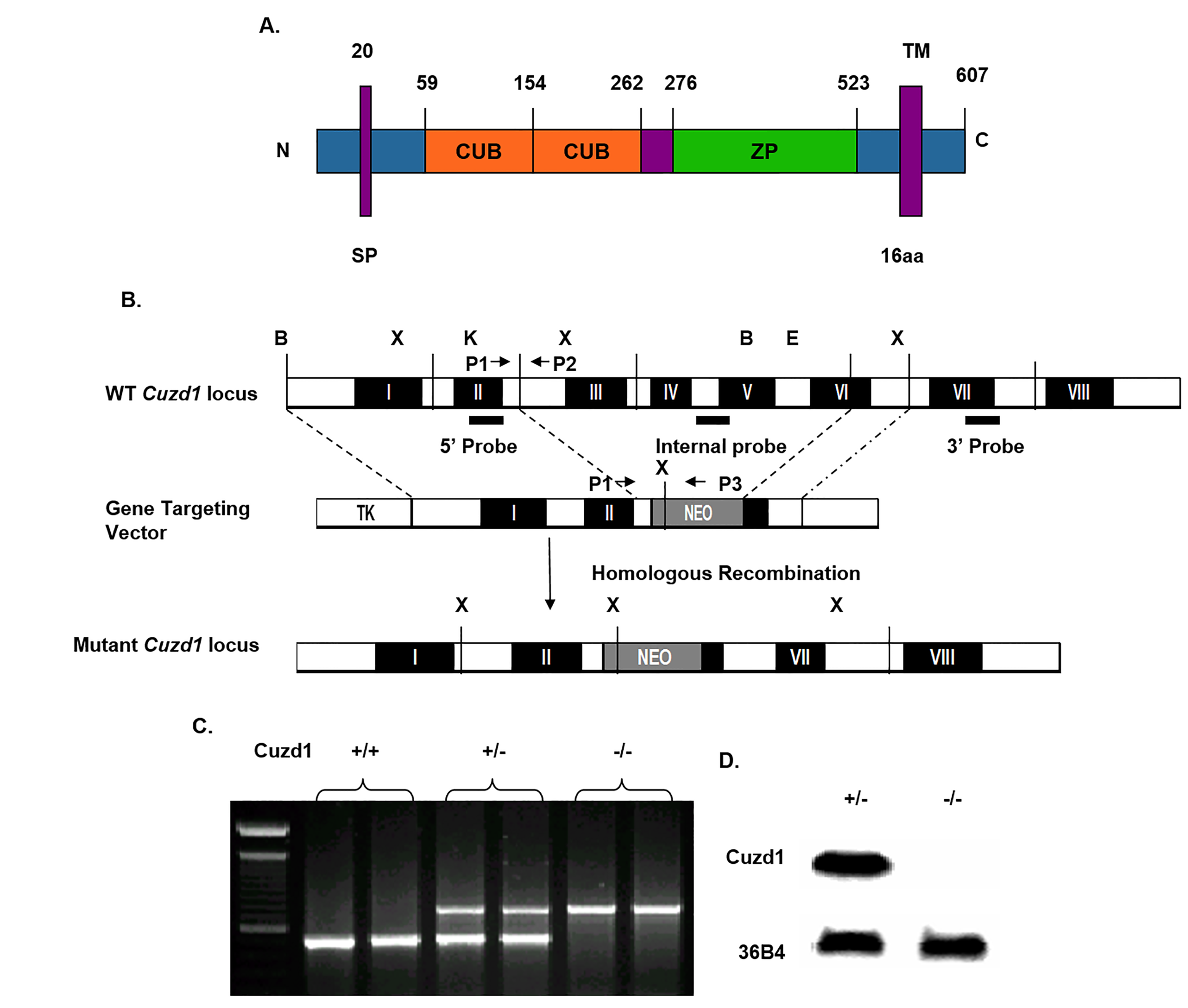

Supplement: S1 Fig — (A) Protein structure of CUZD1. CUZD1 contains two tandem CUB (Complement subcomponent /C1s, Uegf, Bmp1) motifs and a zona-pellucida (ZP)-like domain. (B) Map of Cuzd1 target. The genomic organization of the wild-type Cuzd1 allele is shown with black boxes representing exons and white boxes representing introns. In the targeting vector, the neomycin (NEO) resistance gene is included to provide clone selection. Homologous recombination results in replacement of exons III-VI by the NEO resistance gene. P1, P2, P3 represent the locations of primers used for genotyping PCR to identify wild-type or null genomic mutation. A subset of restriction enzyme sites is shown for relative orientation and targeting vector construction: B, BamHI; E, EcoRI; X, XhoI. (C) Genotyping of Cuzd1-null mice. Genotyping was performed by PCR using tail genomic DNA as template and P1 and P2 or P1 and P3 as primers. The 513 bp and 782 bp DNA fragments arose from wild-type and mutant loci, respectively. (D) Measurement of Cuzd1 mRNA. Total RNA was isolated from pregnant (day 1) uteri of heterozygous Cuzd1(+/-) and homozygous Cuzd1(-/-) mice. The RNA was subjected to Northern blotting, using P32-labled probes specific for Cuzd1 and internal control gene, 36B4. (+/+), (+/-), and (-/-) represent genomic DNA of wild-type, heterozygous and homozygous mice, respectively. (TIF) [file pgen.1006654.s001.tif]

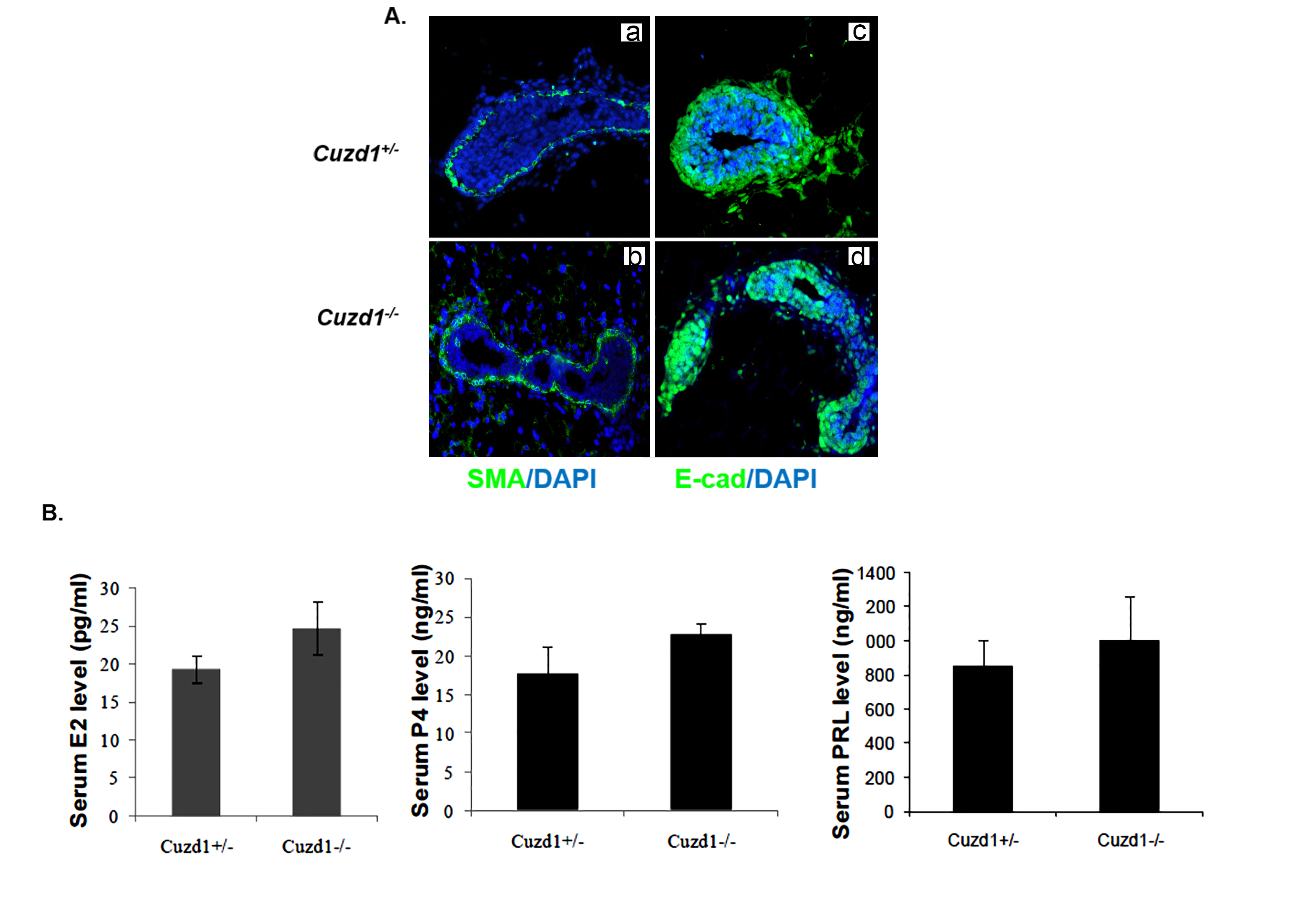

Supplement: S2 Fig — (A) Expression of SMA and E-cadherin in terminal end buds. Mammary tissue sections obtained from Cuzd1(+/-) and Cuzd1(-/-) mice were subjected to IHC using an antibody specific for SMA (a and b) and E-cadherin (c and d). (B) Hormone level measurements. Blood samples were collected from heterozygous Cuzd1(+/-) and homozygous Cuzd1(-/-) mice on day 18 of pregnancy. The measurements of E, P and PRL levels were performed as described in Experimental Procedures. Bars represent average values ± SEM from five animals of each genotype. (TIF) [file pgen.1006654.s002.tif]

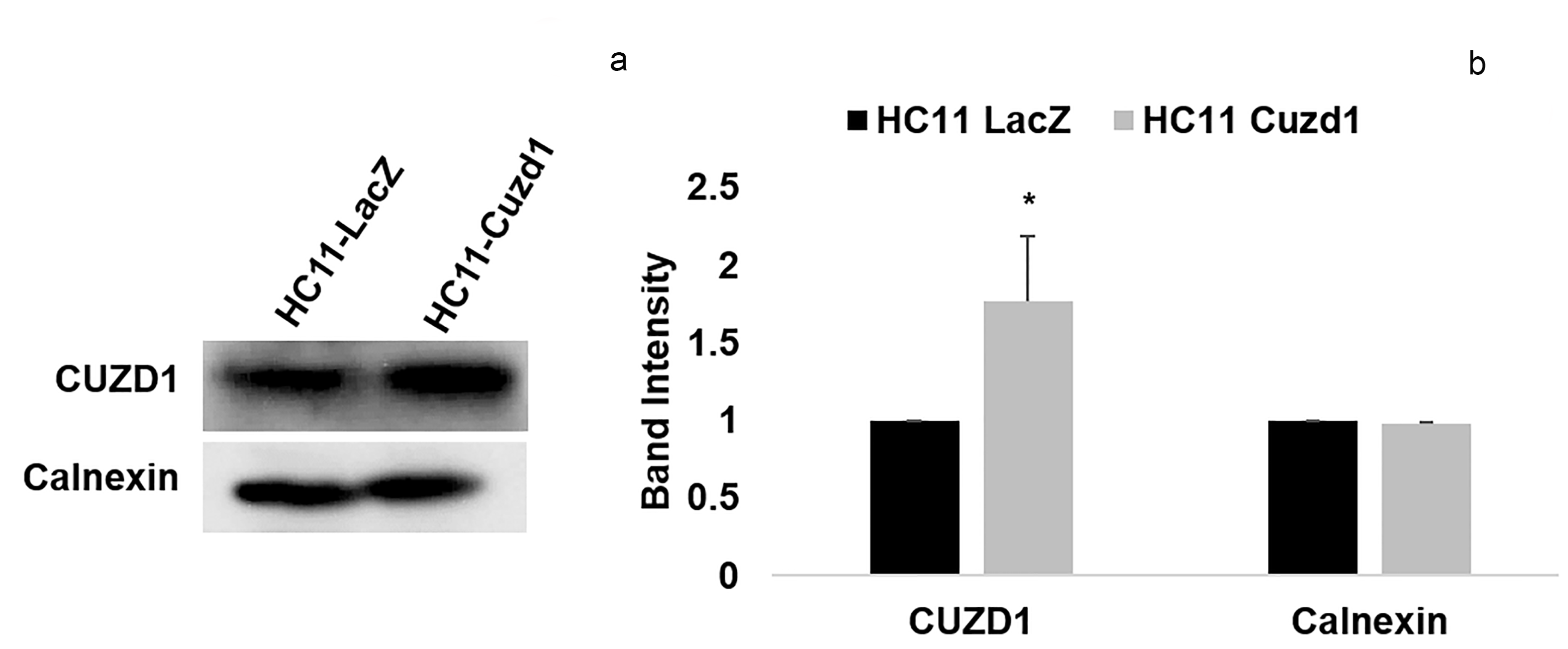

Supplement: S3 Fig — HC11 cells were transduced with lentivirus harboring Cuzd1 or LacZ cDNA to create stable cells overexpressing Cuzd1 (HC11-Cuzd1) or LacZ (HC11-LacZ), respectively. HC11-LacZ and HC11-Cuzd1 cells were lysed and total protein extracts were analyzed using Western blot. Blots were probed with an antibody specific for CUZD1. Calnexin was used as a loading control (a). The density of the bands was quantified using ImageJ and are expressed as Band Intensity (b). (TIF) [file pgen.1006654.s003.tif]

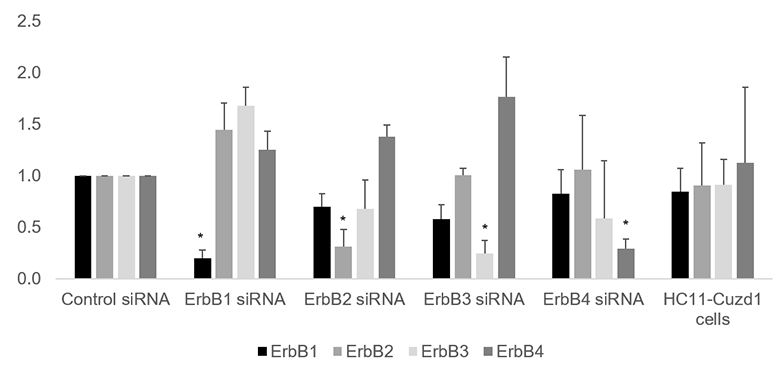

Supplement: S4 Fig — HC11-Cuzd1 cells were transfected with siRNA (50nM) targeted against ErbB1, ErbB2, ErbB3, ErbB4 or scrambled siRNA (control). Total RNA was isolated from these cells and subjected to real-time PCR using specific primers to validate ErbB 1–4 mRNA expression. Data are represented as relative gene expression ± SEM. (TIF) [file pgen.1006654.s004.tif]

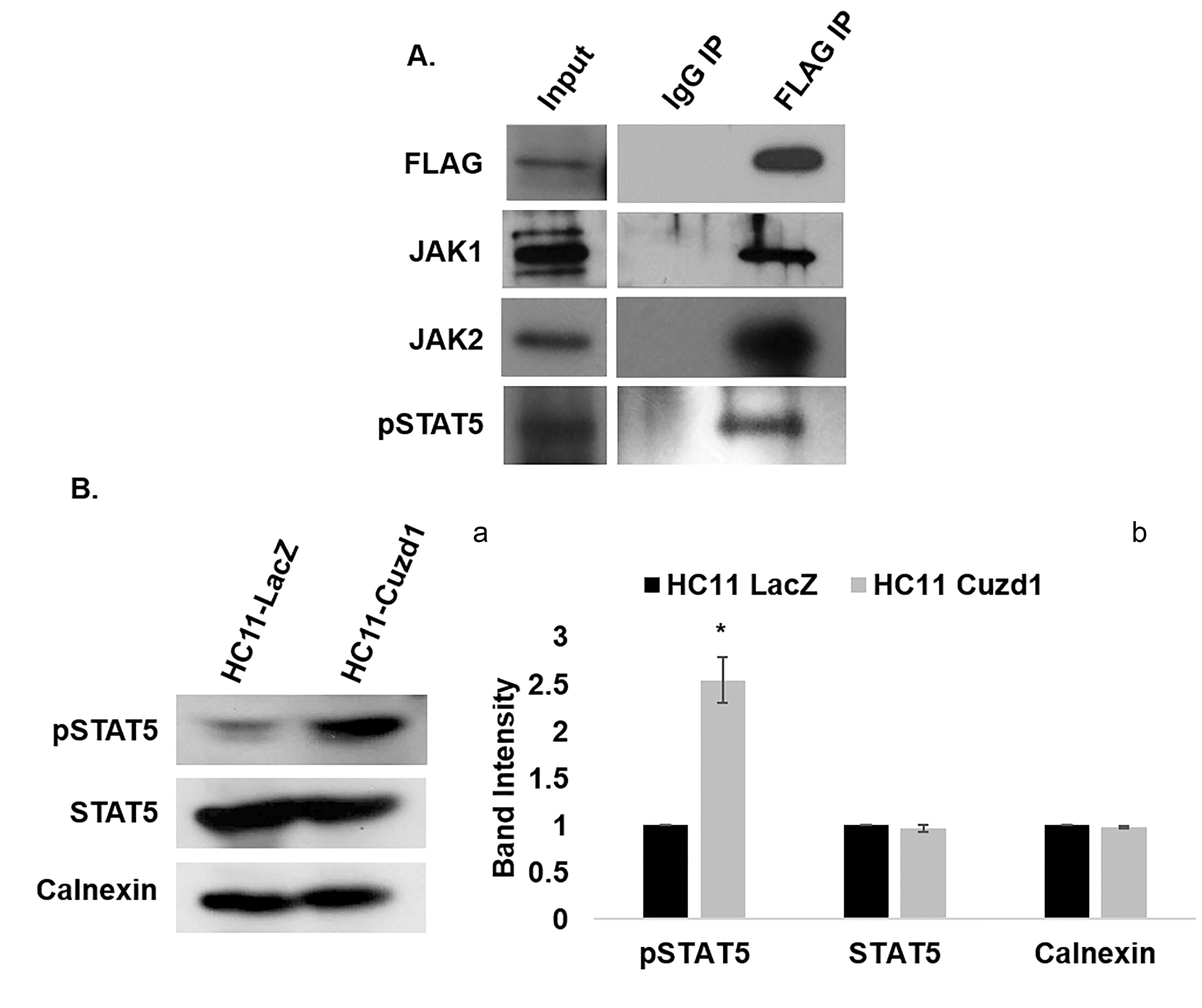

Supplement: S5 Fig — (A) Confirmation of CUZD1 protein complex in HC11-3xFLAG-Cuzd1 cells. HC11-3xFLAG-Cuzd1 cells were cultured for 48 h in serum-free media and then treated with FBS/PRL/EGF for 6 h. Cells were lysed and samples were immunoprecipitated with an IgG (control) or M2 (anti-FLAG) antibody. 3xFLAG-CUZD1 and the associated proteins were confirmed by Western blot analysis. Blots were probed with FLAG, JAK1, JAK2 and pSTAT5 antibodies. (B). Alteration in STAT5 phosphorylation in Cuzd1 overexpressing cells. HC11-LacZ and HC11-Cuzd1 cells were lysed and total protein extracts were analyzed using Western blot. Blots were probed with an antibodies specific to STAT5 and pSTAT5. Calnexin was used as a loading control (a). The density of the bands was quantified using ImageJ and are expressed as Band Intensity (b). (TIF) [file pgen.1006654.s005.tif]

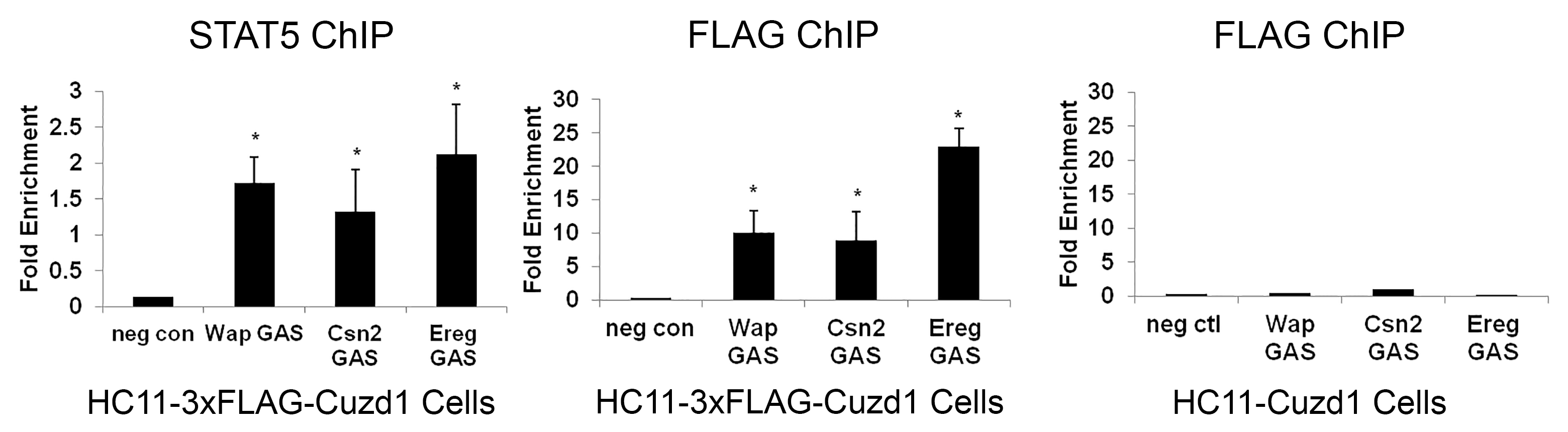

Supplement: S6 Fig — HC11-3xFLAG-Cuzd1 and HC11-Cuzd1 cells were cultured with FBS/EGF/PRL for 6h. Protein/DNA complexes were precipitated using an antibody for STAT5 or FLAG, and subjected to qPCR using primers specific to GAS motifs of Wap, Csn2, and Ereg promoters, respectively. Data are represented as relative gene expression ± SEM. (TIF) [file pgen.1006654.s006.tif]
