# Supplementary material for: CUZD1 is a critical mediator of the JAK/STAT5 signaling pathway that controls mammary gland development during pregnancy
Source: PLoS Genet. 2017 Mar 9;13(3):e1006654. doi: 10.1371/journal.pgen.1006654 (PMC5363987; doi:10.1371/journal.pgen.1006654)
Supplement: S1 Text — (DOCX) [file pgen.1006654.s007.docx]

**MATERIALS AND METHODS**

**Reagents**

pKO Scrambler NTKV-1901 targeting vector was purchased from Stratagene (La Jolla, CA). G418, carmine, formalin, hyaluronidase and 1.5 mg/ml collagenase, insulin, p3XFLAG-CMV-10 Expression Vector, progesterone and 17β-estradiol, mouse IgG-agarose, ANTI-FLAG M2 affinity resin, 3xFLAG peptide were purchased from Sigma-Aldrich (St. Louis, MO). JAK1, JAK2, EREG, Ki67, pErbB1 (Tyr 1068), pErbB2 (Tyr 877), pErbB4 (Tyr 1056), pSTAT5 (Tyr694) antibodies were obtained from Santa Cruz Biotechnology (Dallas, TX). The NRG1 antibody was purchased from Thermo Scientific (Waltham, MA). Total STAT5, EPGN, ErbB1, ErbB2 and ErbB4 antibodies were acquired from Cell Signaling Technology (Beverley, MA). Horseradish peroxidase (HRP)–conjugated goat anti-mouse or goat anti-rabbit secondary antibodies, plenti6.3/V5 TOPO Trizol RNA purification kit, RPMI-1640, blasticidin, Prolong GOLD antifade reagent with 4′,6-diamidino-2-phenylindole, Cuzd1 siRNA and non-targeting siRNA and Lipofectamine-RNAimax were purchased from Life Technologies (Carlsbad, CA). Cyanine 3 or Dylight 488-conjugated antimouse IgG or antirabbit IgG were obtained from The Jackson Laboratory (Bar Harbor, ME). Fetal bovine serum was purchased from Atlanta Biologicals (Atlanta, GA). Prolactin was acquired from the National Hormone and Peptide Program (Torrance, CA).
