# Supplementary material for: CUZD1 is a critical mediator of the JAK/STAT5 signaling pathway that controls mammary gland development during pregnancy
Source: PLoS Genet. 2017 Mar 9;13(3):e1006654. doi: 10.1371/journal.pgen.1006654 (PMC5363987; doi:10.1371/journal.pgen.1006654)
Supplement: S1 Table — (DOCX) [file pgen.1006654.s008.docx]

| **Gene symbol** | **Gene accession** | **Forward primer** | **Reverse primer** |
| --- | --- | --- | --- |
| Cuzd1 | NM_008411 | GCAGCAGGTGTGAAACTGAA | GATTCAAACACAGGCACGAA |
| Wap | NM_011709 | AACATTGGTCTTCCGAAAGC | AGGGTTATCACTGGCACTGG |
| Csn2 | NM_009972 | TCCTCTCTTGTCCTCCACTA | TGTAGCATGATCCAAAGGTGA |
| krt19 | NM_008471 | CTGCTGTCTGGCAATGAGAA | CGAGGCACTCAAGGAAGAAC |
| 36B4 | NM_007475.3 | CATCACCACGAAAATCTCCA | TTGTCAAACACCTGCTGGAT |
| Epgn | NM_053087 | CGAAGAAGCAGAGGTGATCC | AATGGCTTGCTTCAGCTCAT |
| Ereg | NM_007950 | CTACACTGGTCTGCGATGTGA | TCCAGCGGTTATGATGAGAAAC |
| Areg | NM_009704 | AGATGTCTTCAGGGAGTG | GGTATTTGTGGTTCGTTATC |
| Egf | NM_010113 | TTCTCACAAGGAAAGAGCATCTC | GTCCTGTCCCGTTAAGGAAAAC |
| Btc | NM_007568 | TGAAAACCCACTTCTCTCGGT | TGCTGGAGGTAAAACAGGTCC |
| Nrg1 | NM_178591 | TCAGCAAGTTAGGAAACGACAG | ACATAAGGTCTTTCAGTTGAGGC |
| Nrg2 | NM_001167891 | ACGGATTCTTCGGACAGAGAT | CACAGGACACTTTGCTTAGGAT |
| Nrg3 | NM_001190187 | TAGGCTCCGTCAAGGAGTACG | GGGGACGTGGTAGAAGTGG |
| Nrg4 | NM_032002 | CACGCTGCGAAGAGGTTTTTC | CGCGATGGTAAGAGTGAGGA |
| Hbegf | NM_010415 | AGATACCTGCAGGAGTTCCG | GTCATAACCTCCTCTCCTGT |
| Wap GAS |  | CATCTCTTCCTGCCCATGAC | TCGGGCATACATTGAAAAGG |
| Csn2 GAS |  | GTCCTCTCACTTGGCTGGAG | GTGGAGGACAAGAGAGGAGGT |
| Ereg GAS |  | GCGAATTGCATCCTGTGAGT | ACCCCCTCACATTTTGGAGA |
